# Hypertension: Development of a prediction model to adjust self-reported hypertension prevalence at the community level

**DOI:** 10.1186/1472-6963-12-312

**Published:** 2012-09-11

**Authors:** Graciela Mentz, Amy J Schulz, Bhramar Mukherjee, Trivellore E Ragunathan, Denise White Perkins, Barbara A Israel

**Affiliations:** 1 Department of Health Behavior and Health Education, School of Public Health, University of Michigan, Ann Arbor, MI, USA; 2 Department of Biostatistics, School of Public Health, University of Michigan, Ann Arbor, MI, USA; 3 Institute of Multicultural Health, Henry Ford Health System, and Department of Family Practice, Henry Ford Hospital, Detroit, MI, USA

## Abstract

**Background:**

Accurate estimates of hypertension prevalence are critical for assessment of population health and for planning and implementing prevention and health care programs. While self-reported data is often more economically feasible and readily available compared to clinically measured HBP, these reports may underestimate clinical prevalence to varying degrees. Understanding the accuracy of self-reported data and developing prediction models that correct for underreporting of hypertension in self-reported data can be critical tools in the development of more accurate population level estimates, and in planning population-based interventions to reduce the risk of, or more effectively treat, hypertension. This study examines the accuracy of self-reported survey data in describing prevalence of clinically measured hypertension in two racially and ethnically diverse urban samples, and evaluates a mechanism to correct self-reported data in order to more accurately reflect clinical hypertension prevalence.

**Methods:**

We analyze data from the Detroit Healthy Environments Partnership (HEP) Survey conducted in 2002 and the National Health and Nutrition Examination (NHANES) 2001–2002 restricted to urban areas and participants 25 years and older. We re-calibrate measures of agreement within the HEP sample drawing upon parameter estimates derived from the NHANES urban sample, and assess the quality of the adjustment proposed within the HEP sample.

**Results:**

Both self-reported and clinically assessed prevalence of hypertension were higher in the HEP sample (29.7 and 40.1, respectively) compared to the NHANES urban sample (25.7 and 33.8, respectively). In both urban samples, self-reported and clinically assessed prevalence is higher than that reported in the full NHANES sample in the same year (22.9 and 30.4, respectively). Sensitivity, specificity and accuracy between clinical and self-reported hypertension prevalence were ‘moderate to good’ within the HEP sample and ‘good to excellent’ within the NHANES sample. Agreement between clinical and self-reported hypertension prevalence was ‘moderate to good’ within the HEP sample (kappa =0.65; 95% CI = 0.63-0.67), and ‘good to excellent’ within the NHANES sample (kappa = 0.75; 95%CI = 0.73-0.80). Application of a ‘correction’ rule based on prediction models for clinical hypertension using the national sample (NHANES) allowed us to re-calibrate sensitivity and specificity estimates for the HEP sample. The adjusted estimates of hypertension in the HEP sample based on two different correction models, 38.1% and 40.5%, were much closer to the observed hypertension prevalence of 40.1%.

**Conclusions:**

Application of a simple prediction model derived from national NHANES data to self-reported data from the HEP (Detroit based) sample resulted in estimates that more closely approximated clinically measured hypertension prevalence in this urban community. Similar correction models may be useful in obtaining more accurate estimates of hypertension prevalence in other studies that rely on self-reported hypertension.

## Background

Hypertension affects an estimated 30% [1-3] of the population in the United States, and is associated with health outcomes such as cardiovascular disease, heart attack and stroke [4-8]. Population estimates of hypertension prevalence are often assessed through large scale surveys which rely on participant self reports of previous clinical diagnosis of hypertension [5,9]. Self-reported data is often more economically feasible and readily available (e.g., through telephone interviews [10,11]) compared to clinically measured high blood pressure (HBP). However, given substantial evidence that awareness of hypertension is imperfect (for example, discrepancies between clinical measures and self-reported hypertension), reliance on self-reported data may contribute to inaccuracies in estimating population prevalence of hypertension [12-15]. Furthermore, given evidence that awareness varies across various subgroups within the United States [16-19], reliance on self-reported data to estimate prevalence in small areas where population characteristics differ from national characteristics may contribute to inaccuracies in prevalence estimates.

Several studies have examined the validity of self-reported hypertension and its use for surveillance of hypertension trends. Studies using national data such as NHANES [18,20] or large samples [11,21,22] have suggested that self-reported data may underestimate hypertension prevalence [10,12-15], given that some with hypertension are unaware or otherwise do not report the condition [5,16,23]. Age, gender, education, geographic area, marital status, race and ethnicity have been found to be associated with accuracy of self-reported HBP [4,6,7,16,24-27]. Studies that have attempted to gauge the extent of this problem have reported differences between clinically measured and self-reported HBP that range from 2.0 [5] to 27.0% [16]. Most studies designed to assess the accuracy of self-report data have compared self-reported high blood pressure to a ‘gold standard’ [17,23,28-31] such as measurements obtained from physical examinations using a mercury sphygmomanometer [26,32]. The majority of these studies have been based on small samples; have relied on volunteers; include only persons in good health; or recruit participants of particular organizations (e.g., an HMO) or screening programs. These factors limit the ability to either generalize to broader populations or identify characteristics that may be associated with differential accuracy of the self-reported versus clinically measured HBP. One validation study has been based on a nationally representative sample [33], and this study identified a prediction model used to estimate prevalence of high blood pressure. These methods were developed for large-scale national samples, and require fairly sophisticated statistical expertise to implement.

However, there are well-established differences in the rates, awareness and treatment of hypertension across racial and ethnic groups, by socioeconomic status, and across geographical areas within the United States [25,34]. Thus, the applicability of national models within specific communities or areas may vary. In addition, the severity of the underestimation of self-reported data varies across different chronic diseases [16,23] such as diabetes, stroke and heart attacks [11,35-38]. Assessing the validity of self-reported data in estimating hypertension prevalence in specific geographic areas, and developing simple prediction models that correct for possible miss reporting of HBP in self-reported data, can be essential to the creation of accurate population level estimates, and for population level efforts to effectively prevent or treat HBP within particular contexts. To date, no studies of which we are aware have developed such a correction model for self-reported data at local geographic levels.

Thus, our objective in this paper is to examine the accuracy of self-reported data in describing the prevalence of hypertension in racially and ethnically diverse urban community, and to develop a simple tool to correct self-reported data to more accurately reflect clinical prevalence of HBP. Specifically, we aim to:

Aim1: Examine the extent to which reliance on self-reported data may miss-characterize hypertension prevalence in a multiethnic urban community.

Aim2: Develop a prediction model to calibrate self-reported data to more closely correspond to the clinical prevalence of hypertension in a local community sample.

To address these aims, we draw on data from two multiethnic urban samples, the 2002 Healthy Environments Partnership (HEP) community survey [39] and the NHANES 2001–2002 national survey, restricted to residents 25 years and older of metropolitan areas as described in the following section.

## Methods

### Sample and data collection

Data for this study are drawn from two sources: 1) the Healthy Environment Partnership (HEP) community survey conducted in 2002 with adults aged 25 and older living in three areas of Detroit, Michigan; and 2) the NHANES 2001–2002 data, restricted to the subset of the sample collected in urban metropolitan Careas, and 25 years of age and older, in order to match the HEP sample.

The HEP survey is a two-stage probability sample of occupied housing units (households), designed for 1,000 completed interviews of adults, age 25 years and older. The complex design features allow for comparisons of residents of similar demographics across geographic areas of the city. The final study sample consisted of 919 valid face-to-face interviews completed in 2002. Interviews were completed with 75% of households in which an eligible respondent was identified [39]. Data was collected included self-reported demographic variables, psychosocial, behavioral, and socioeconomic indicators, and both self-reported and clinically measured BP.

The NHANES 2001–2002 sample is a nationwide probability sample of the population selected via a complex series of statistical techniques (for references on the design of the NHANES sample refer to http://www.cdc.gov/nchs/nhanes/about_nhanes.htm). For this study we restrict the NHANES 2001–2002 sample to include cases that have clinical measured hypertension (n = 4878). For this sample to closely match the HEP sample we limited the national sample to include only urban metropolitan areas and individuals 25 years old and older (n = 1114). We refer to this sample henceforth as the NHANES urban sample.

### Measures

#### Dependent variables

The dependent variable for this study was high blood pressure (HBP), and we included both self-report and clinically measured indicators within each sample.

*Self-reported high blood pressure* status (SR) in both HEP and the NHANES studies, was determined based on the response to the question “Has a doctor or other health provider ever told you that you had hypertension, also called high blood pressure?” Individuals who responded “yes” to this question were coded as having self-reported high blood pressure.

*Clinically measured high blood pressure*, (CH) was constructed as follows, for both the HEP and the NHANES survey data. Three measures of systolic and diastolic blood pressure were obtained using a portable cuff device (Omron model HEM 711 AC) that passed Association for the Advancement of Medical Instrumentation standards [40]. In both the mean of the second and third reading were calculated for systolic and diastolic blood pressure readings. CH was defined as mean systolic blood pressure > 140 and/or mean diastolic blood pressure >90 and/or self-report of current treatment with antihypertensive medication.

#### Independent variables

Independent variables included in the prediction models were derived from survey data and included age in years; gender (ref: female); marital status (ref: married); annual household income categorized into four levels: less than $10 K, $10 K-$19 K, $20 K-$34 K and $35 K or more (ref); and education categorized into three levels: less than 12 years of education, completed high school and more than 12 years of education (ref); self-reported race and ethnicity, categorized as Hispanic, non-Hispanic White and non-Hispanic Black (ref). Non-Hispanic Black was chosen as the reference group because it’s the largest group size for the HEP sample, and affords the most robust standard errors for the re-calibration step of the process.

#### Statistical analysis

The statistical analysis for this paper has two distinct parts, each one corresponding to one of the two aims of the paper described in the previous section: Aim 1) Assessment of concordance between self-reported and clinically measures HBP was performed for both samples (HEP and NHANES urban samples); Aim 2) Calibration of measures of agreement within the HEP sample drawing upon parameter estimates derived from the NHANES urban sample.

#### Assessment of validity and concordance between self-reported and clinically measures HBP (Aim 1)

Statistical measures of 1) *sensitivity* (percent fitting the medical criteria for hypertension who reported that they had the condition); 2) *specificity* (percent not fitting the medical criteria who reported they do not have the condition); 3) *accuracy* or *overall level of agreement* (percent for whom the medical criteria and self-reported are in agreement); and 4) *Cohen’s Kappa* coefficient with adjustment for chance agreement were used to assess the validity and concordance between of self-reported and clinical measure hypertension within each urban sample (HEP and NHANES urban).

Each statistical measure was calculated for the full sample, and also stratified by each of the independent variables considered for this analysis (e.g. age, gender, race and ethnicity, education and household income).

#### Calibration of sensitivity and specificity within the HEP sample drawing upon parameter estimates derived from the NHANES urban sample (Aim 2)

Our objective is to develop a simple prediction model to calibrate self-reported data to more closely correspond to the clinical prevalence of hypertension in a local community sample. To do so we drew form the NHANES urban sample described above. The NHANES urban sample was stratified by whether a participant reported having ever been told by a doctor that they had HBP, with “yes” coded as SR = 1 and “no” coded as SR = 0, respectively. We then fitted weighted logistic regression models within each strata using IVEWare %Regress procedure (SAS Windows 9.2) to predict each individual’s probability of having clinically measured HBP adjusting for age, gender, marital status, race and ethnicity, indicators of annual household income, and education.

The parameter estimates from these two logistic models were then applied (‘plug-in method’) to the HEP sample to obtain re-calibrated estimates of sensitivity and specificity for the HEP sample, for each participant as follows:

(1) $$\text{specify}=1-\hat{P}\left(CH_{i}=1|SR_{i}=0,X_{i}\right)=1-\frac{\text{exp}\left(X_{i}{\hat{\beta }}_{o}\right)}{1+\text{exp}\left(X_{i}{\hat{\beta }}_{o}\right)}$$

(2) $$\text{sensitivity}=\hat{P}\left(CH_{i}=1|SR_{i}=1,X_{i}\right)=\frac{\text{exp}\left(X_{i}{\hat{\beta }}_{1}\right)}{1+\text{exp}\left(X_{i}\;{\hat{\beta }}_{1}\right)}$$

Where ${\hat{\beta }}_{o}$ and ${\hat{\beta }}_{1}$ are the vectors of point estimates from the two logistic models fitted within each of the strata of the NHANES urban sample, SR =1 (been told they have HBP) or SR = 0 (never told have HBP).

We considered two approaches to obtain estimates of the probability of clinically measured HBP within the HEP dataset. These are described below as Method 1 and 2.

Method 1:

a) For individuals who reported never being told they had HBP (SR = 0), we estimate the probability of having clinically measured HBP using one minus the specificity estimate described in Equation (1).

b) For individuals who reported having been told they had HBP (SR = 1), we estimate the probability of having clinically measured HBP using the sensitivity estimate described in Equation (2).

Method 2: This alternate method is relevant when one wants to estimate marginal prevalence of clinically measured HBP for individuals by weighting Method 1 estimates with estimated probability of self-reported HBP. This method may be sought if self-reported data is not complete or reliable and one wants to impute/replace it by using covariate information.

a) Estimates of the probability of self-reported HBP within the HEP sample, using weighted logistic regression models adjusting for the same set of covariates used to develop the prediction model described in Method 1.

(3) $$\hat{P}\left(CH_{i}=1|SR_{i},X_{i}\right)$$

b) Estimate of the probability of clinically measured HBP as a weighted average of re-calibrated sensitivity and specificity as follows:

(4) P^CHi=1|Xi=P^CHi=1|SRi=0,Xi*P^SRi=0|Xi+P^CHi=1|SRi=1,Xi*P^SRi=1|Xi

where $\hat{P}\left(SR_{i}=0|X_{i}\right)=1-\hat{P}\left(SR_{i}=1|X_{i}\right)$

Finally, we then considered 0.50, 0.60, 0.70 and 0.80 as threshold values of the estimated probability of clinically measured HBP which were used to classify each individual into one of two groups: HBP according to clinical measures or not (binary representation of predicted probabilities based on both proposed methods). That is if,

(5) $$\hat{P}\left(CH_{i}=1|X_{i}\right)>\text{threshold},$$

we classify the HEP participant as having predicted clinically HBP, i.e.,

(6) $${\hat{CH}}_{i}=1,$$

otherwise we classify him/her as not having predicted clinically HBP. For each proposed threshold misclassification rates were assessed using measures of

(7) $$\text{Sensitivity}=P\left(\hat{CH}=1|CH=1\right),$$

and

(8) $$\text{Specificty}=1-P\left(\hat{CH}=1|CH=0\right),$$

where $\hat{CH}$ and CH are predicted and known clinically measured HBP within the HEP sample respectively, using weighted cross-classification techniques. The threshold value with lowest miss-classification rate was proposed for final classification.

## Results

As shown in Table 1, the average age of HEP community survey participants was 46.3±0.8 years, 52.3% were female, 56.8% non-Hispanic Black, 22.6% Hispanic (of this group, 75.8% identified as Mexican American); 37.3% had less than 12 years of education, 27.3% had household incomes less than $10,000, 26.4% were married, and the mean number of household members was 2.8. For the NHANES urban sample the average age was 48.2±0.4 years, 52.1% were female, 10.5% were non-Hispanic Black; 12.3% Hispanic (with 84.7% of this group identified as Mexican American), 19.0% had less than 12 years of education, 18.3% had household incomes less than $10,000, 62.4% were married, and the mean number of household members was 3.7.

**Table 1:** HEP and NHANES demographic measures

		**HEP**^**1**^	**NHANES**^**2**^
		**(N = 919)**	**(N = 1114)**
Age, mean (stddev^3^)		46.3(0.8)	48.2(0.4)
Age, %	25-34	46.1	46.3
	35-49	33.6	34.4
	50+	20.3	19.3
Gender, %	Female	52.3	52.1
Marital Status, %	Married	26.4	62.4
Race/Ethnicity, %	Latino	22.2	12.3
	non-Hispanic White	18.8	72.7
	non-Hispanic Black	56.8	10.5
	Other	2.3	4.5
Education, %	<12 years	37.3	19.0
	12 years	29.5	25.0
	>12 years	33.2	56.0
Annual Household Income^4^, %	<$10,000	27.3	18.3
	$10,000-19,999	26.0	15.5
	$20,000-34,999	23.6	21.6
	$35,000+	23.0	44.6
Number of members in HU, mean(stddev)		2.8(0.1)	3.7(0.1)

1: Healthy Environments Partnership.

2: National Health and Examination Survey restricted age 25+ and Urban.

3: Stddev = Standard deviation.

Prevalence of hypertension based on self-reported data underestimates the clinical prevalence by 10.4% for the full HEP sample (Table 2). When we consider prevalence estimates for different demographics indicators such as age (continuous and categorical), gender, race and ethnicity, education and annual household income, underestimates range from 7.3% to 13.9%. In particular, the largest percent of under-reporting were observed for non-Hispanic Whites (13.9%) and for those with annual household income between $20 K-$34 K (13.6%).

**Table 2:** Prevalence of self-reported and clinically measured HBP by levels of the covariates included in the prediction models (HEP1 sample)

	**N**	**HBP**^**2**^**Prevalences**		**Difference**^**3**^
		**Clinical**	**Self-reported**	
Overall	919	40.1	29.7	10.4
Age				
25-34	242	15.6	8.2	7.4
35-49	342	34.3	23.3	11.0
50+	335	64.8	52.5	12.3
Gender				
Female	632	39.2	31.9	7.3
Male	287	41.1	27.2	13.9
Marital Status				
Current Married	230	38.3	27.5	10.8
Not Married	689	49.5	37.8	11.7
Race/ethnicity				
Latino	182	26.6	16.0	10.6
non-Hispanic White	199	41.2	27.3	13.9
non-Hispanic Black	522	46.0	36.3	9.7
Education				
<12	327	43.5	31.4	12.1
12	259	38.3	28.9	9.4
12+	321	37.8	28.8	9.0
Annual Household Income				
<$10,000	250	47.7	40.4	7.3
$10,000-19,999	238	39.4	29.7	9.7
$20,000-34,999	230	37.3	23.7	13.6
$35,000+	201	34.7	22.8	11.9

1: Healthy Environments Partnership.

2: Hypertensive if mean systolic BP= >140 or mean diastolic BP= > 90 or currently taking hypertensive medication.

3: Difference between clinical and self-reported hypertension.

Sensitivity, specificity, accuracy (overall agreement) and Kappa statistics of the self-reported measure of HBP for the full HEP sample and stratified by each investigated determinant are shown in Table 3. These results show that self-reported and clinical measure hypertension for the HEP sample have generally ‘moderate to good’ agreement: sensitivity (range = 0.77-0.97); specificity (range = 0.77-0.83); accuracy (range = 0.81-0.83); and overall Kappa ( range = 0.65-0.66). Comparing Tables 2 and 3, we conclude that the use of self-reported data has “good” validity, and is likely to underestimate population based hypertension prevalence within the HEP sample.

**Table 3:** Sensitivity, specificity and agreement indicators for each determinant investigated (age, gender, marital status, educational level, income level)

	**Sensitivity**	**Specificity**	**Accuracy**^**1**^	**Kappa**
	**P(CH = 1|SR = 1)**	**P(CH = 0|SR = 0)**	**(overall agreement)**	
Full sample	0.90	0.80	0.83	0.66
Age				
25-34	0.71	0.88	0.85	0.64
35-49	0.86	0.81	0.82	0.57
50+	0.95	0.69	0.83	0.83
Gender				
Females	0.88	0.83	0.85	0.66
Males	0.9	0.8	0.8	0.59
Marital Status				
Currently Married	0.90	0.82	0.84	0.65
Not Married	0.89	0.80	0.83	0.65
Race/ethnicity				
Latino	0.89	0.85	0.86	0.65
non-Hispanic White	0.90	0.77	0.81	0.65
non-Hispanic Black	0.90	0.79	0.83	0.66
Education				
<12	0.92	0.79	0.83	0.65
12	0.79	0.82	0.81	0.65
12+	0.86	0.82	0.83	0.66
Annual Household Income				
<$10,000	0.77	0.83	0.81	0.66
$10,000-19,999	0.92	0.83	0.86	0.65
$20,000-34,999	0.89	0.79	0.81	0.65
$35,000+	0.97	0.77	0.83	0.66

Accuracy is the proportion of true results (both true positive and true negative). Refer to Additional file 1.

Tables 4 and 5 show that the under- reporting of hypertension using self-reported data in the NHANES national urban sample is generally smaller than in the HEP sample (range = 6.1%-11.5%). Within this sample, validity and concordance are generally in the ‘good to excellent’ range: sensitivity(range = 0.89-0.99); specificity (range = 0.82-0.89); accuracy (range = 0.86-0.90); and overall Kappa ( range = 0.75-0.77). Thus, we propose the use of the national urban sample to develop the prediction models described in Aim 2.

**Table 4:** Prevalence of self-reported and clinically measured HBP by levels of the covariates included in the prediction models (NHANES1 urban sample)

	**N**	**NHANES**^**2**^**Prevalences**		**Difference**^**3**^
		**Clinical**	**Self-reported**	
Overall	1124	33.8	25.7	8.1
Age				
25-34	172	37.4	30.6	6.8
35-49	256	61.1	54.7	6.4
50+	169	67.0	51.5	15.5
Gender				
Females	583	34.3	28.2	6.1
Males	541	33.2	22.9	10.3
Marital Status				
Current Married	692	31.6	23.3	8.3
Not Married	432	37.9	30.1	
Race/ethnicity				
Latino	261	21.1	10.8	10.3
non-Hispanic White	614	35.8	27.7	8.1
non-Hispanic Black	215	40.3	31.1	9.2
Education				
<12	366	45.5	36.4	9.1
12	251	36.7	27.7	9.0
12+	502	28.2	20.9	7.3
Annual Household Income				
<$10,000	166	46.9	35.4	11.5
$10,000-19,999	177	44.2	36.3	7.9
$20,000-34,999	221	37.0	29.0	8.0
$35,000+	307	30.8	22.5	8.3

1: NHANES urban sample.

2: Hypertensive if mean systolic BP= >140 or mean diastolic BP= > 90 or currently taking hypertensive medication.

3: Difference between clinical and self-reported hypertension.

**Table 5:** Sensitivity, specificity and agreement indicators for each determinant investigated (sex, educational level, income level) (NHANES Urban Sample N = 1114)

	**Sensitivity**	**Specificity**	**Accuracy**	**Kappa**
	**P(CH = 1|SR = 1)**	**P(CH = 0|SR = 0)**	**Overall agreement)**	
Full sample	0.92	0.86	0.88	0.77
Age				
25-34	0.93	0.87	0.89	0.78
35-49	0.99	0.70	0.83	0.67
50+	0.99	0.68	0.84	0.60
Gender				
Females	0.94	0.89	0.90	0.75
Males				
Marital Status				
Current Married	0.91	0.86	0.88	0.76
Not Married	0.91	0.84	0.86	0.77
Race/ethnicity				
Latino	0.99	0.88	0.90	0.77
Non-Hispanic White	0.92	0.86	0.87	0.74
Non-Hispanic Black	0.93	0.84	0.87	0.76
Education				
<12	0.96	0.83	0.88	0.75
12	0.93	0.85	0.87	0.76
12+	0.90	0.88	0.88	0.76
Annual Household Income				
<$10,000	0.96	0.82	0.87	0.76
$10,000-19,999	0.93	0.89	0.90	0.76
$20,000-34,999	0.89	0.85	0.86	0.77
$35,000+	0.90	0.89	0.90	0.77

Accuracy is the proportion of true results (both true positive and true negative). Refer to Additional file 1.

In order to avoid overestimation of prediction models, sample sizes should be at least 15–30 per predictor [41-43]. Stratified sample sizes within the NHANES urban sample were insufficient to meet this threshold, and therefore would have compromised the stability of the prediction model. Thus, we used the full NHANES urban sample to develop the prediction model.

In Table 6, we show parameter estimates based on weighted logistic models for predicting clinically measured HBP from self-reported HBP, using the NHANES urban sample. Results are shown stratified by the self-report indicator, that is, with separate models and parameter estimates for those who reported having been told, versus never having been told by a health care provider that they had HBP. These parameter estimates were then applied (‘plug-in’ method) to the HEP sample to obtain re-calibrated estimates of sensitivity and specificity for each HEP participant as indicated by Equations (1) and (2) above. Self-reported HBP for each HEP participant was also estimated. Finally, the probability of clinically measured HBP was then obtained using both Methods 1 and 2 (described above).

**Table 6:** Coefficients of prediction models of clinical hypertension stratified by self-report using NHANES urban sample

	**Clinical HBP**^**1**^	
	**Self-reported = Yes**	**Self-reported = NO**
	**Estimate (StErr)**	**Estimate (StErr)**
Intercept	−0.3(1.36)	−2.52(0.6)
Age^2^	0.12(0.03)	0.04(0.01)
Gender		
Females	0.41(0.64)	−0.52(0.21)
Males (reference)	1	1
Marital Status		
Married	−0.36(0.61)	0.04(0.25)
Not Married (reference)	1	1
Race/ethnicity		
Latino	*	−0.46(0.42)
White	0.03(0.84)	−0.43(0.31)
Black (reference)		
Education		
<12	0.51(1.36)	0.02(0.27)
12	0.67(0.84)	0.2(0.29)
12 + (reference)		
Annual Household Income		
<$10,000	0.20(0.99)	0.53(0.37)
$10,000-19,999	−0.48(1.09)	−0.04(0.33)
$20,000-34,999	−0.75(0.65)	0.4(0.51)
$35,000 + (reference)		

1: Clinical measured hypertension is the outcome.

2: Continuous age was used in prediction models.

We then dichotomized these estimates using 0.5, 0.6, 0.7 and 0.8 as threshold values as indicated by Equations (5) and (6) above. In Table 7 we present estimates of sensitivity and specificity of the binary representation of the predicted probability with respect to the available measure of clinical HBP (Equations (7) and (8)) for each of the threshold values. For both methods 1 and 2, the greatest sensitivity and specificity are found for the threshold value of 0.50 At this threshold level, the overall final adjusted estimate of prevalence of HBP was 38.1% (sensitivity = 0.90; specificity = 0.78) for Method 1 and 40.5% (sensitivity = 0.92; specificity = 0.79) for Method 2. Both final adjusted estimates were considerably closer to the clinically derived prevalence of 40.1% (Table 2) for the HEP community sample, compared to the unadjusted self report estimate of 29.7%.

**Table 7:** Comparison of predicted and measured high blood pressure for different threshold values (HEP sample)

**Threshold (p)**^**3**^	**Method 1**^**1**^		**Method 2**^**2**^	
	**Sensitivity Pr(CH = 1|SR = 1)**	**Specificity Pr(CH = 0|SR = 0)**	**Sensitivity Pr(CH = 1|SR = 1)**	**Specificity Pr(CH = 0|SR = 0)**
0.5	0.90	0.78	0.92	0.78
0.6	0.89	0.75	0.91	0.77
0.7	0.85	0.66	0.90	0.76
0.8	0.79	0.62	0.89	0.74

1: Method 1.

a) For individuals who reported never being told they had HBP (SR = 0), we estimate the probability of having clinically measured HBP using one minus the specificity estimate described in Equation (1).

b) For individuals who reported having been told they had HBP (SR = 1), we estimate the probability of having clinically measured HBP using the sensitivity estimate described in Equation (2).

2: Method 2.

a) Estimates of the probability of self-reported HBP within the HEP sample, using weighted logistic regression models adjusting for the same set of covariates used to calculate sensitivity and specificity.

b )Estimate of the probability of clinically measured HBP as a weighted average of re-calibrated sensitivity and specificity.

3: Threshold values of the estimated probability of clinically measured HBP which were used to classify each individual into one of two groups, Clinical Hypertensive or not.

## Discussion

Findings reported here suggest that self-reported data underestimate the prevalence of high blood pressure in the NHANES urban sample by 8.1% and in the HEP local community sample by 10.4%. These underestimates are larger than those reflected in the full NHANEs 2001–2002 sample of 7.5%, suggesting that the degree of underestimation of hypertension prevalence based on self-reported data may be larger in urban compared to national samples. Furthermore, prevalence of hypertension appears to be higher in the two community samples used in this analysis (33.8% and 40.1% for the NHANES urban and HEP samples respectively), compared to the 30.4% reported for the NHANES 2001–2002 full sample. These results suggest that the application of national rates, or the use of corrections derived from national samples, may not be appropriate to estimate hypertension prevalence in some urban communities.

We found the highest levels of HBP, and the greatest discrepancies between self-reported and clinically measured HBP, in the HEP community sample. This community is characterized by a higher proportion of residents with lower socioeconomic status, and by a greater proportion of Hispanic and non-Hispanic Black participants compared to the NHANES urban sample. The higher levels of HBP, and the greater discrepancy between self-reported and clinically measured HBP in this sample may reflect more restricted access to health care providers compared to the NHANES urban or the NHANES national sample.

Our finding are consistent with results reported in the literature when considering large samples, simulations or national sample such as the NHANES sample. We extend these by showing the under-estimation of hypertension for self-reported data can be even larger in small communities. The range of the underreporting for the HEP sample of 7.3% to 13.9% fell within the range of 0.2% to 27% reported in other studies that have assessed the validity and concordance of self-reported data when considering small samples like the one used in this paper. The wide range of under-reporting complicates the generalization of findings from one community to another. Thus, developing a prediction model that will allow re-calibration of self-reported data for small samples seems reasonable and appropriate.

Our second aim was to examine the feasibility of using prediction models to correct for underestimates of prevalence of HBP using self-reported data. The application of prediction models derived from the NHANES urban sample to data from the HEP community sample resulted in re-adjusted estimates of sensitivity and specificity. These adjusted estimates were then used to obtain improved estimates of the probability if hypertension that more closely correspond to clinically measured levels of HBP in this community. Final re-calibrated estimates of hypertension, 38.1% and 40.5% for the HEP sample, using both Method 1 (Equations (1) and (2)) and Method 2 (Equations (3) and (4)) resulted in estimates that were much closer to population prevalence of hypertension, 40.1%. These findings suggest that prediction models similar to those used here can be applied to obtain more accurate estimates of hypertension prevalence in local communities.

In this case, we created and applied a prediction model based on national metropolitan (as an approximation of urban) data to a local multi-ethnic urban community. Based on the findings reported here, we suggest that prediction models can be used to adjust self-report HBP data to obtain more accurate estimates of HBP prevalence by following the procedures described below:

1. Using NHANES 2001–2002 data similar to the researcher’s data set, predict the probability of clinical hypertension stratified by self-reported hypertension, i.e., using SR_i_ and X_i_ Apply the coefficient estimates of the logistic models into the researcher’s sample (in our case the HEP sample) to re-calibrate sensitivity and specificity estimates using Equations (1) and (2).

2. Estimate the probability of self-report HBP using Equation (3) for each participant.

3. Estimate the probability of clinically measured HBP using Equation (4) for each participant.

4. Using the threshold value of 0.50 classify each respondent into one of two groups: has clinically measures HBP or not.

### Limitations

Like most studies, there are a number of limitations that should be considered in interpreting the findings reported here. The comparison between NHANES 2001–2002 urban sample and the HEP community sample is limited by a number of factors. There are important differences between the NHANES urban and the HEP sample in racial and ethnic composition, income and education, each of which are important correlates of high blood pressure in the United States. While we have adjusted for these factors in our models, it is feasible that these differences in the structure of the samples may have influenced the findings reported here.

## Conclusions

Finding presented here reiterate the importance of developing them means to handle self-reported data developing disease specific and community specific models. The accuracy of self-report of HBP prevalence differs from the accuracy of self-report for other diseases (citations) and varies across communities. Simple models like the ones proposed in this paper are easy to implement and can be a very important tool to re-calibrate self-reported data to better estimate chronic disease prevalence for local communities.

Despite the limitations described above, the findings reported here suggest that the use of prediction models may be useful in creating estimates of hypertension prevalence based on self-report data. Differences were larger in the Detroit based community sample, which also had the highest rates of HBP (regardless of type of measure) suggesting that reliance on self-report data may disproportionately underestimate prevalence of HBP in low to moderate income, racially and ethnically diverse urban communities such as Detroit.

Our results indicate that reliance solely on measures of agreement to determine validity of self-reported data in small samples whose demographic characteristics differ from those of national samples may be conducive to underestimation of hypertension prevalence. While a number of studies using large national samples have reported validity of self-reported data based on measures of sensitivity and specicity [18,20-22], the findings reported here suggest that in smaller, more localized samples, the use of prediction models that account for the mischaracterization of self-reported data jointly with measures of agreement may result in more accurate estimates of hypertension prevalence. The relatively simple prediction models proposed here provided a re-calibrated prevalence of hypertension estimate that more closely corresponded to the clinical hypertensive prevalence for the Detroit sample to which it was applied in this example.

The non-stratified prediction models used in this example improved the accuracy of overall estimates of prevalence of HBP derived from self-reported data, which is much less costly to collect than clinically measured HBP. As a result, such prediction models offer a low cost approach to improve prevalence estimates and thus the ability to plan prevention and treatment efforts to reduce high blood pressure and its negative health effects. Given limited funds available for public health surveillance, health promotion and treatment efforts, prediction models that enable accurate estimates at lower costs may allow limited funds to be shifted toward health promotion and treatment efforts in high-risk urban populations.

## Competing interests

The authors declare that they have no competing interests.

## Authors' contributions

GM participated in the conception of the study, carried out the statistical analysis and drafted the manuscript. AS participated in the conception of the study, and helped to draft the manuscript. BM helped with the identification of the appropriate statistical analysis and helped draft the manuscript. TR helped with conception of the analysis, supervised the statistical analysis.BI helped to draft de manuscript. DWP helped draft the manuscript. All authors read and approved the final manuscript.

## Pre-publication history

The pre-publication history for this paper can be accessed here:

http://www.biomedcentral.com/1472-6963/12/312/prepub

## Supplementary Material

Additional file 1 Accuracy is the proportion of true results (both true positive and true negative). Click here for file
