# Supplementary material for: Hypertension: Development of a prediction model to adjust self-reported hypertension prevalence at the community level
Source: BMC Health Serv Res. 2012 Sep 11;12:312. doi: 10.1186/1472-6963-12-312 (PMC3483283; doi:10.1186/1472-6963-12-312)
Supplement: Additional file 1 — Accuracy is the proportion of true results (both true positive and true negative). [file 1472-6963-12-312-S1.doc]

Additional file 1 Accuracy is the proportion of true positive results (both true positive and true negative)

| Acuracy range | Classification |  |  |  |
| --- | --- | --- | --- | --- |
| 0.9<Accuracy<1.0 | Excellent |  |  |  |
| 0.8<Accuracy<0.9 | Good |  |  |  |
| 0.7<Accuracy<0.8 | Worthless |  |  |  |
| 0.6<Accuracy<0.7 | Not good |  |  |  |
| Kappa range | Classification |  |  |  |
| <0.20 | Poor agreement |  |  |  |
| 0.20 to 0.40 | Fair agreement |  |  |  |
| 0.40 to 0.60 | Moderate agreement | |  |  |
| 0.60 to 0.80 | Good agreement |  |  |  |
| 0.80 to 1.00 | Very good agreement | |  |  |
